# The Burden of Bacteriologically Negative TB Diagnosis: A Four-Year Review of Tuberculosis Cases at a Tertiary Facility

**DOI:** 10.1155/2023/6648137

**Published:** 2023-12-23

**Authors:** Jane S. Afriyie-Mensah, Robert Aryee, Francisca Zigah, Ernest Amaning-Kwarteng, Marie Nancy Séraphin

**Affiliations:** ^1^Department of Medicine and Therapeutics, University of Ghana Medical School, University of Ghana, Accra, Ghana; ^2^Department of Physiology, University of Ghana Medical School, University of Ghana, Accra, Ghana; ^3^Department of Cardiology, University of Ghana Medical Centre, Accra, Ghana; ^4^Korle-Bu Teaching Hospital, Accra, Ghana; ^5^Emerging Pathogens Institute, University of Florida, 2055 Mowry Road, P.O. Box 100009, Gainesville, FL 2610, USA; ^6^Department of Medicine, Division of Infectious Diseases and Global Medicine, College of Medicine, University of Florida, College of Medicine, Gainesville, FL 32610, USA

## Abstract

**Aim:**

We aimed to investigate the demographic and clinical factors associated with TB mortality in patients managed at a tertiary TB referral center.

**Methods:**

We conducted a retrospective review of the medical records of 1,933 TB patients seen between January 2017 and December 2020 at the Korle-Bu Teaching Hospital (KBTH) Chest Department in Accra, Ghana. TB mortality was defined as any TB patient who died for any reason during the course of treatment. Multivariable logistic regression was used to estimate adjusted odds ratios with 95% confidence intervals for factors associated with TB mortality.

**Results:**

A total of 1,933 patients with TB were registered at the chest clinic over the study period. Males accounted for 1,227 (63.5%), and majority of participants were between 24 and 64 years old. Pulmonary TB (PTB) and extrapulmonary TB (EPTB) cases accounted for 51% and 48.4% of the total TB cases, respectively. A significant proportion (69%) of the patients managed for TB had no bacteriological confirmation of the disease. About 34% of tested TB patients were HIV positive. Mortality among patients was 33.6%. In a multivariable regression model, patients with HIV positive status had over 3-fold increased risk of mortality, compared to those with HIV negative status. TB patients diagnosed empirically had an increased risk of death compared to those with a confirmed diagnosis.

**Conclusion:**

The proportion of clinically diagnosed TB was high among the patients seen at the chest clinic. Mortality was high among the patients with HIV/TB coinfection as well as in patients with empirical TB diagnosis.

## 1. Introduction

Tuberculosis (TB) is an infectious disease of public health concern caused by *Mycobacterium tuberculosis* (Mtb). The disease has a global presence with an incidence of about 10.6 million cases per annum but is more prevalent in low- to middle-income countries [1]. TB is the leading cause of infectious disease-related mortality globally. According to the World Health Organization [1, 2], more than 80% of global TB mortality occurs in low- to middle-income countries. TB case reports in Africa account for a quarter of the global incidence of TB and also contribute over 25% of the global mortality caused by TB [2]. A survey by Law et al. in 12 African countries showed that TB deaths in Africa alone contribute about 32% to the global TB deaths [3]. The high prevalence of TB in Africa has been attributed to the increased prevalence of HIV on the continent, particularly in sub-Saharan Africa [4], and this is supported by the finding that majority (84%) of HIV/TB coinfection cases worldwide reside in sub-Saharan Africa [5]. The prevalence of TB in Ghana is considered high with an estimated figure of 290 per 100,000 population as per the 2013 survey [6]. The country is also listed as one of the 30 countries with a high TB/HIV burden [1]. In 2019, 44,000 people were estimated to have had TB in Ghana [5].

TB can affect every organ in the body but predominantly the lungs, causing pulmonary TB (PTB) in about 85% of all cases. Extrapulmonary TB (EPTB), which is defined as involvement of other parts of the body aside the lungs, has been increasing over recent years with variable prevalence globally, particularly among HIV positive patients [7–9]. The diagnosis of TB is confirmed by the presence or isolation of Mycobacterium tuberculosis (Mtb) bacilli in a biological specimen (sputum, other body fluids, or biopsy material) either by microscopic examination with the Ziehl-Neelsen (ZN) staining, nucleic acid amplification testing (NAAT), culture, or histological examination. Mycobacterium culture which is the gold standard significantly improves the diagnosis of TB among suspected but smear/NAAT negative TB cases. However, culture capacity is scarce in resource poor countries coupled with the time consuming limitation of the test [10]. Depending on culture results to diagnose TB leads to treatment delays and poor treatment outcomes. WHO currently recommends initial rapid molecular diagnostic tests such as the GeneXpert® MTB/RIF for patients with signs and symptoms suggestive of TB, and this technology, through the global fund, has been distributed to most low- to middle-income countries though limited in number. Smear microscopy which is cheap but less sensitive (about 60% sensitivity) for the diagnosis of TB is still being used in resource-limited settings [10–12]. Extrapulmonary TB (EPTB) is a complex disease with a paucibacillary nature and may therefore require invasive procedures to obtain tissue for diagnosis. Where a high clinical suspicion of TB exists without a positive bacteriological isolation in a sample or suggestive histology, WHO recommends a presumptive diagnosis of TB and prompt initiation of therapy especially among HIV and suspected EPTB patients [8].

The use of GeneXpert® MTB/RIF to detect Mtb DNA has proven to have a much higher detection rate when compared to smear microscopy and has a diagnostic agreement of >90% with culture, which is regarded as the gold standard test [11–13]. It is therefore expected that GeneXpert will significantly improve diagnosis of pulmonary TB (PTB) or mixed PTB/EPTB cases globally and reduce the proportion of bacteriology unconfirmed or presumed TB cases [12–14]. Globally, it is estimated that 57% of TB cases are bacteriologically confirmed, and this proportion increases to approximately 80% in resource-rich countries where a plethora of other investigative measures exists to confirm the diagnosis and is accessible to majority of their citizens [15]. The burden of presumed TB cases remains high in most resource-limited countries such as occurs in sub-Saharan Africa but lack of data makes it difficult to objectively quantify the problem. In a study in Gambia, about 50% of the patients with presumed TB diagnosis did not actually have the disease but found later to have treatable alternate diagnoses despite having chronic respiratory symptoms [16]. A systematic review by Jayasooriya et al. [17] estimated the proportion of clinically presumed TB cases to be 48.5% in passive case findings and could be as high as 93% in active TB case search strategies [17]. The reasons for this observation include lack of widespread distribution of GeneXpert machines in resource-limited countries, hence the significant reliance on the less sensitive smear microscopy. Secondly, confirming TB diagnosis in smear/GeneXpert negative suspected TB cases may require extensive investigations such as bronchoscopy to obtain BAL, image-guided biopsy or aspiration from extrapulmonary disease sites for histology, and advanced radiological imaging (MRI and CT scan) of specific affected organs. These investigative measures are not only scarce in the above settings but expensive and not readily accessed by most suspected TB patients. The experienced physician in such jurisdictions is therefore encouraged to make a presumed clinical diagnosis of TB based on suggestive signs and symptoms in the majority of cases. This well-meaning action to prevent treatment delays due to the aforementioned diagnostic challenges could also wrongly presume TB diagnosis resulting in poor treatment outcomes as alternative diagnoses are obscured and left untreated. The most affected are HIV patients and patients with suspected EPTB. Unnecessary exposure to TB medications with increased adverse effects and deaths is another major effect of wrong TB diagnosis. There is therefore the possibility that some recorded TB deaths attributed to TB may be erroneous and could bloat estimates of TB mortality in countries with significant proportions of presumed TB cases [17–19]. It is thus crucial to improve objective TB diagnosis, and inability to increase proportion of confirmed TB cases (either bacteriological confirmation or suggestive histology/laboratory tests) could stifle WHO target of reducing TB deaths by 95% in 2035 [20].

Our study was conducted in a low- to middle-income country with a high TB and TB/HIV prevalence. Anecdotal evidence suggests an increasing number of clinically presumed TB diagnoses in a major TB referral hospital. This study therefore sought to describe the clinical profile and associated risk factors for all-cause mortality in TB patients diagnosed and treated at the hospital's chest department over a four-year period.

## 2. Methods

### 2.1. Study Setting

The chest clinic of the Korle-Bu Teaching Hospital is a major tertiary facility in Accra, Ghana, for the comprehensive care of adolescent and adult TB patients. The clinic has a functional outpatient department (OPD), in-patient wards for TB cases requiring admission, a well-resourced mycobacteriology lab, pharmacy, public health unit, directly observed therapy (DOT) service, voluntary and counseling testing (VCT) unit, digital X-ray unit, and nutritional counseling services. All cases of TB in the hospital, except children with TB, are seen and managed at the chest clinic. It is a major TB referral site for the Greater Accra Region and other parts of the country and also receives referrals from the West Africa subregion.

### 2.2. Design and Study Population

The study was a retrospective review of the medical charts of all adolescent and adult patients (≥13years) diagnosed and treated for TB at the chest clinic of the Korle-Bu Teaching Hospital, from January 2017 to December 2020. Patients referred to the unit on account of suspected TB but not diagnosed or managed for TB after clinical evaluation were not included in the study. A standardized data abstraction tool was used to collect clinical presentation and treatment outcome information from the medical records. Information available from the medical records included age, sex, sputum smear status, Xpert MTB/RIF results, HIV status, chest radiography findings, and site of TB disease. We abstracted the outcome of the prescribed TB treatment regimen from the TB treatment cards.

### 2.3. Variables and Definitions

#### 2.3.1. Patient Type

WHO definitions were used to classify patients into new TB cases and retreatment cases. The retreatment group included patients who resumed TB therapy after a period of loss to follow-up (defaulted patients), as well as those who at the end of the most recent treatment course were declared cured or treatment complete. New TB patients were those who had never been treated for TB or had received less than one month of anti-TB treatment [8].

#### 2.3.2. Tuberculosis Diagnosis

Screening and diagnosis of TB follow the national TB algorithms, where individuals with TB symptoms are first screened with an X-ray followed by bacteriological testing (smear microscopy or the Xpert MTB/RIF assay using the GeneXpert system). All suspected TB patients presenting to the chest clinic are offered voluntary counseling and testing for HIV. Diagnosis of TB was based on clinical presentation, X-ray/CT scan findings, and bacteriology (smear or GeneXpert results), where available. Clinically diagnosed TB/presumed TB cases are those who did not meet the criteria for confirmed TB diagnosis with a bacteriology negative specimen or suggestive histology but was prescribed the full course of TB treatment based on clinical considerations alone or suggestive radiological image [8].

#### 2.3.3. Tuberculosis Disease Site

We classified as PTB all cases with TB involving the lungs, either bacteriologically positive or negative. EPTB was defined as TB disease involving anatomical sites other than the lungs, and these were categorized based on the organ system affected, e.g., pleura, lymph nodes, meninges, joints and bones, or spine. For the purposes of this study, we defined disseminated TB as TB disease involving two or more noncontiguous organ systems aside the lungs.

#### 2.3.4. Tuberculosis Treatment

Patients diagnosed with drug-susceptible TB were initiated on the recommended TB regimen comprising of two-month intensive treatment with four drugs, rifampicin (€), isoniazid (H), pyrazinamide (Z), and ethambutol (E), followed by at least four more months of RH. Sputum smear microscopy was repeated for all patients especially those with pulmonary TB at the end of months two and five of treatment as well as upon completion.

#### 2.3.5. Tuberculosis Treatment Outcome

Treatment outcome was documented based on WHO definition and classified as cured, treatment completed, treatment failure, death, or loss to follow-up [8]. Bacteriologically confirmed TB patients who completed the full treatment course with a negative smear at months two and five and at the end of therapy were classified as cured. Those who completed therapy without evidence of treatment failure but no record of a smear or culture report were classified as treatment complete. For the study, “loss to follow-up” was defined as patients who interrupted their therapy for at least two consecutive months. Patients who failed therapy were those whose sputum smear remained positive at five months or upon completion of treatment.

### 2.4. Data Analysis

All data were collected in Microsoft Excel and imported into SPSS version 24 for analysis. Statistics such as mean, standard deviation, and frequencies was used to describe the study population. The chi-square test was used to determine associations between the categorical variables. We fitted bivariate and multivariable logistic regression models to measure crude (OR) and adjusted odds ratio (aOR) with 95% confidence interval (CI) for the association of clinical and demographic risk factors with TB mortality in the study population. *P* value < 0.05 was considered statistically significant.

### 2.5. Ethical Consideration

Data abstracted from the medical records were uniquely coded with no direct means of linking it back to the individual patient. Patient data captured on Excel was stored in a password-protected folder on a dedicated research laptop kept in a research office accessed only by the research team. Ethical review and approval for the study was obtained from the Korle-Bu Hospital institutional review board (IRB).

## 3. Results

### 3.1. Characteristics of the Study Participants

The baseline demographic and clinical characteristics of the cases are presented in Table 1. A total of 1,933 patients with a diagnosis of TB were registered at the Korle-Bu Teaching Hospital over the study period. Males accounted for 1,227 (63.5%) of the population and most were within the 24–64 age bracket. The majority (94%) of the cases seen were new TB cases, and the proportions of PTB and EPTB were 51.01% and 48.37%, respectively. In general, about 31% (606/1933) of the total TB cases had bacteriological confirmation while 138 (7.1%) had no documentation of a bacteriology report. From the results, the proportion of PTB cases with positive bacteriology was 606/986 (62%); about 25% was smear/GeneXpert negative, and 126 (13%) had no documentation of bacteriology status. Regarding EPTB cases, none of those tested had a positive sputum test, and for the remainder, there were no documented suggestive histology or laboratory records such as pleural adenosine deaminase. HIV status was known in 1672 (86.5%) of the patient population with 576 (34.4%) testing positive. Those who refused HIV testing were 261 (13.5%). The proportion of HIV patients diagnosed with EPTB and PTB was 281 (48.8%) and 291 (50.5%), respectively, and only about 26% of the HIV patients had bacteriology confirmation of TB.

About a quarter (25.6%) of all the TB cases diagnosed at the chest clinic were transferred out to treatment facilities closer to patients' residence to improve accessibility of care, and hence, their treatment outcomes were unavailable for analysis. Treatment outcome for the 1,438 patients who continued to receive care at the chest clinic was as follows: 752 (52.3%) successfully completed treatment, 483 (33.6%) died, 154 (10.7%) defaulted on treatment, and 7 (0.48%) were documented as treatment failure (Table 1).

The different EPTB disease sites seen in our study population are presented in Table 2. EPTB cases were predominantly tuberculous pleural effusion and disseminated TB accounting for 34% and 37%, respectively. Pott's disease or TB of the spine accounted for 12% of the EPTB cases.

### 3.2. Demographic and Clinical Factors Associated with TB Mortality

From Table 3, 218 (45.1%) and 225 (46.6%) of those who died had HIV and EPTB, respectively, and about 20% of the deaths had bacteriological confirmation of TB while 63% had no such confirmation. The results of the univariate and multivariable logistic regression are presented in Table 3. The positive association between age at diagnosis and TB mortality was not statistically significant as well as no significant association between gender and TB mortality (*P* = 0.246). The site of TB infection was also not significantly associated with TB mortality, although the mortality among PTB cases (52.8%) was slightly higher than that among EPTB cases (46.6%). In a multivariable regression model, HIV infection was associated with an over 3-fold increased risk of mortality, compared to patients who were HIV negative. TB patients with no bacteriology confirmation (unconfirmed status) were more likely to die compared to patients with bacteriological confirmation of TB (*P* = 0.023). The association was however attenuated in a fully adjusted model (OR = 0.981, 95% CI = 0.680-1.415).

## 4. Discussion

The cohort of TB patients reviewed was largely male, which has been observed in several studies involving TB patients [21–23]. The male-to-female ratio was estimated to be 1.6 : 1 by WHO although variable across countries [24]. Tuberculosis is associated with significant socioeconomic impact as majority of those affected are within their reproductive age groups and this was similarly observed in this study with the majority (46%) of patients aged 25–44 years followed by patients in the 45–64 age group (31%). Globally, there have been reports of some reduction in the incidence of PTB but rather a trend of increasing prevalence of EPTB has been observed with variable rates in different populations [25, 26]. The estimated global prevalence of EPTB in 2020 was 16% of all TB cases, but in sub-Saharan Africa, EPTB cases were shown to have exceeded 30% of reported new and retreatment cases of TB in a 2017 report by WHO [24]. The current study also found a high prevalence of EPTB, with almost half (48%) of the TB population diagnosed with EPTB. This was higher than reported rates of 29% and 20-30%, respectively, in previous local studies [21, 22]. A similar study in a referral hospital in Ethiopia reported an EPTB proportion of 49.8% [27]. Being a major TB referral site could explain the high proportion of EPTB as the latter tends to have significant diagnostic challenges, and hence, patients are likely to be referred [27, 28]. Notable among the risk factors, driving the increasing prevalence of EPTB is HIV/AIDS [29, 30]. Thus, the high prevalence of EPTB in the current study could also be explained by the fact that a significant proportion of the TB cohort had HIV (34%).

With respect to the WHO report, the global proportion of TB cases with bacteriology confirmation is about 57% but this proportion tends to be much higher in resource-rich settings compared to low-resource areas [15]. The reasons for this picture are multiple including the significant lack of diagnostic work-up for suspected but bacteriology negative TB cases. Bacteriology negative TB cases describe PTB or EPTB cases in which the obtained biological specimen is negative for AFBs on microscopy, GeneXpert, or culture. However, TB diagnosis can still be confirmed in the absence of a bacteriologically positive specimen, and this occurs when a patient has clinical features that are highly suspicious of TB and has a suggestive histology or laboratory report such as pleural ADA levels [8]. Lack of bacteriological confirmation of TB is a major problem as it increases the possibility of wrongly diagnosing and treating TB, subsequently leading to poor treatment outcomes [31]. The increased risk of adverse effects from the unwarranted TB treatment is also a threat to the health of such patients [32].

The prevalence of clinically diagnosed TB is believed to be on the rise in sub-Saharan Africa, but the magnitude and burden have not been well assessed and could be one of the reasons for the persistently high TB mortality in the subregion [17, 33]. The systematic review by Jayasooriya et al. demonstrated a high burden of presumed TB cases, ranging from 48.5% to 92.8% depending on a passive or an active case search approach [17]. In regions known to have a high burden of TB, particularly in developing countries, the policy priority has been to improve TB case detection and early initiation of treatment targeted at reducing associated morbidity and mortality. This approach from the systematic review has a tendency of increasing diagnosis of bacteriology negative TB cases [17]. In low resourced and TB endemic countries, a significant proportion of patients with chronic respiratory symptoms and an abnormal CXR have a higher chance of being diagnosed as smear/GeneXpert negative PTB [34]. This is portrayed in a study at a TB clinic in Gambia, where about 52% (125) of the patients were bacteriology negative based on molecular tests and 86.4% (108) of these patients were found to have alternate diagnoses other than TB. Similarly, in a community survey in Malawi, only 10-20% of those with chronic cough had TB, yet due to lack of resources to conduct further investigations, most of these could have been empirically treated for PTB [35]. In total, only about 31% of the TB cohort reviewed in the current study had positive bacteriology specimen, far less than the WHO global estimate of 57% [15]. The study also showed that all the bacteriology positive cases were PTB patients representing about 62% of the total PTB patients in the cohort. Thus, only about 25% of the PTB cases were diagnosed as smear/GeneXpert negative TB while none of the EPTB patients had any documentation of a suggestive histology/laboratory report to confirm the diagnosis of TB. This highlights the diagnostic limitations of EPTB and to a lesser extent PTB [36]. TB pleuritis formed the majority of EPTB cases seen. Analysis of pleural fluid has poor yield on smear/GeneXpert due to its paucibacillary status; thus, supportive biochemical assays such as adenosine deaminase help to secure the diagnosis. This test, although available, is expensive and takes a while to obtain report in our setting. Bouton et al. [37] reviewed 394 retreatment TB cases at the same study site over a period of 6 years and found that for 41% of them, TB was treated presumptively with a mortality rate of 19.4% [37]. In the absence of bacteriological evidence, diagnostic work-up for confirming suspected PTB and EPTB can be a costly and time-consuming venture.

To date, majority of PTB diagnosis is based on direct sputum smear microscopy, culture, and currently molecular tests, but the sensitivity of these is relatively low as a good number of PTB cases are unable to produce sputum or have paucibacillary sputum as seen predominantly in patients co-infected with HIV [38, 39]. This was similarly observed in the current study where only 26% of the HIV patients had a positive sputum test. According to WHO, diagnosis of EPTB can be made based on the basis of a culture positive specimen or a suggestive histology in the setting of strong clinical evidence consistent with TB [15]. Secondly, a combined reference standard including smear, culture, histopathology, and biochemical analysis such as adenosine deaminase levels, response to treatment at 6 months, or suggestive radiological findings is also recommended by WHO, where any positive 2 of the above confirms the diagnosis [15]. In an Ethiopian study by Arega et al., 59.1% of the cases of EPTB had histological evidence and more than one diagnostic method was used for the diagnosis [27]. Another study in Cameroon reported bacteriological confirmation in 41% of the EPTB population. It is worth noting that in both studies, TB lymphadenitis was the most occurring disease which has ease of obtaining lymph node aspirate for analysis. Comparatively, in our setting, the most occurring EPTB cases were pleural TB and disseminated TB. There was heavy reliance on clinical suspicion, mostly after treatment failure of other differential diagnoses and radiological findings. It is therefore not surprising that over 80% of the cases had documentation of suggestive radiological features. There may have been some suggestive histology or biochemical reports in our cohort but missed due to lack of documentation.

The study recorded a high mortality rate, with about a third (33.6%) of the patients with known outcomes dying. A similar tertiary hospital review in Cameroon among HIV positive TB patients reported a mortality rate of 29.4% [40]. Other studies in Malawi and Zimbabwe reported mortality rates of 23% and 22%, respectively [41, 42]. A review by Kebede et al. [43] in Ethiopia also had a lower mortality rate of 24.5% in a hospital-based review of TB cases. Comparatively, the later study had a lower HIV population of 27% with only 18% of them diagnosed with EPTB while our cohort had 34% HIV cases with 49% of them diagnosed with EPTB. These differences could account for the higher mortality in our cohort. Analysis of the patients who died showed that HIV was significantly associated with mortality (*P* < 0.001) and exhibited a 3-fold risk of death compared to HIV negative cases as similarly reported in another studies [33]. Although about 47% of those who died had EPTB diagnosis, this had no significant association with mortality. Being bacteriology unconfirmed was also significantly associated with TB mortality, and this could imply that whether a patient had PTB or EPTB, unconfirmed TB diagnosis is what increases risk of mortality. The significant association was however not sustained on the multivariate analysis. It is worth highlighting the significant mortality observed among the HIV population, the high proportion of EPTB among them (approximately 49%), and the fact that only about a quarter had bacteriological evidence of TB. This scenario questions the surety of TB diagnosis among the HIV patients in particular, and one wonders if other unidentified and untreated diagnoses could have been the cause of death. Delays in establishing the diagnosis of TB in the absence of bacteriological support and other supportive investigations could partly explain the high mortality in this study. Other important factors that contribute to death among HIV patients but not addressed by this study include lack of adherence to ART and TB treatment as well as the duration of symptoms prior to presentation [44–46].

We found that age and gender of the patients did not influence the risk of dying from TB contrary to findings from other studies [47–50]. Although the proportion of TB mortality was slightly higher in males compared to their female counterparts, the difference was not statistically significant. Our findings and earlier reports [21] therefore suggest that gender may not necessarily play a role in TB mortality.

Due to the retrospective design and clinic-based setting of our study, we were limited to working with data previously collected for diagnosis and clinical management, not research. As such, a number of risk factors for TB mortality and important confounders of these associations were not measured.

Although there have been improved diagnosis and treatment programs to reduce TB morbidity and mortality rates globally, TB deaths remain high in sub-Saharan Africa [4]. The need to intensify strategies aimed at reducing TB mortality can therefore not be overemphasized. Effort to confirm TB diagnosis as much as possible in smear/GeneXpert negative PTB cases and EPTB cases is very much needed in our setting. Enhancing precision of TB diagnosis will reduce mortality among suspected TB patients particularly among those with HIV and extrapulmonary sites involvement. Much more important in these categories of patients, where advanced diagnostic measures remain limited, is the need for robust clinical algorithms to increase clinical precision thereby reducing misdiagnosing and the attendant risk of mortality. Comprehensive monitoring of treatment outcomes of clinically diagnosed TB patients could be helpful in the development of such robust symptom-based algorithms. Findings from this study has thrown more light on the need to improve precision in clinical TB diagnosis in both HIV and non-HIV patients and offers a platform for comprehensive review of our diagnostic approach. More efforts are needed to promote VCT among young adults for early diagnosis of HIV to prevent late presentation with advanced TB disease.

## 5. Conclusion

In summary, our study supports the concept that HIV/TB coinfection is associated with increased mortality particularly when TB diagnosis is unconfirmed. Efforts to improve TB confirmation will reduce mortality associated with TB, especially among the HIV population.

## Figures and Tables

**Table 1:** Demographic and clinical characteristics of the study population.

Parameters	Study population *N* (%)	Treatment completed *N* (%)	Lost to follow-up *N* (%)	Died *N* (%)	Treatment failure *N* (%)	Treatment stopped *N* (%)	Transferred out *N* (%)	*P* value
Total sample	1933	752 (38.9)	167 (8.6)	483 (25.0)	7 (0.4)	30 (1.6)	494 (25.6)	

*Age group in years*								
13–24	268 (13.9)	91 (12.1)	23 (13.8)	78 (16.2)	0 (0.0)	3 (10.0)	73 (14.8)	0.524
25–44	890 (46.1)	370 (49.2)	75 (44.9)	217 (44.9)	3 (42.9)	12 (40.0)	213 (43.1)	
45–64	606 (31.4)	228 (30.3)	57 (34.1)	152 (31.5)	3 (42.9)	12 (40.0)	154 (31.2)	
≥65	167 (8.7)	63 (8.4)	12 (7.2)	36 (7.5)	1 (14.3)	3 (10.0)	52 (10.5)	

*Gender*								
Female	706 (36.5)	264 (35.1)	64 (38.3)	186 (38.5)	2 (28.6)	8 (26.7)	182 (36.8)	0.673
Male	1,227 (63.5)	488 (64.9)	103 (61.7)	297 (61.5)	5 (71.4)	22 (73.3)	312 (63.2)	

*Patient type*								
New	1,818 (94.1)	698 (92.8)	157 (94.0)	458 (94.8)	5 (71.4)	29 (96.7)	471 (95.3)	0.382
Retreatment	110 (5.7)	52 (6.9)	10 (6.0)	24 (4.8)	2 (28.6)	1 (4.5)	21 (4.3)	
Not known	5 (0.3)	2 (0.3)	0 (0.0)	1 (0.2)	0 (0.0)	0 (0.0)	2 (0.4)	

*Disease classification*								
Pulmonary	986 (51.0)	424 (56.4)	88 (52.8)	255 (52.8)	5 (71.4)	14 (46.7)	200 (40.5)	<0.001
Extrapulmonary	935 (48.4)	322 (42.8)	78 (46.7)	225 (46.6)	2 (28.6)	16 (53.3)	292 (59.1)	
Not known	12 (0.6)	6 (0.8)	1 (0.6)	3 (0.6)	0 (0.0)	0 (0.0)	2 (0.1)	

*Bacteriological confirmation*								
Confirmed	606 (31.4)	289 (38.4)	58 (34.7)	146 (30.2)	5 (71.4)	0 (0.0)	108 (21.9)	<0.001
Unconfirmed	1189 (61.5)	415 (55.2)	102 (61.1)	300 (62.1)	2 (28.6)	28 (93.3)	342 (69.2)	
No documentation	138 (7.1)	48 (6.4)	7 (4.2)	37 (7.7)	0 (0.0)	2 (6.7)	44 (8.9)	

*Radiological report*								
Suggestive	1,593 (82.4)	628 (83.5)	140 (83.8)	397 (82.2)	5 (71.4)	24 (80.0)	399 (80.8)	0.202
Not suggestive	140 (7.2)	51 (6.8)	12 (7.2)	38 (7.9)	0 (0.0)	2 (6.7)	37 (7.5)	
Not done	102 (5.3)	32 (4.3)	6 (3.6)	26 (5.4)	0 (0.0)	1 (3.3)	37 (7.5)	
Not known	98 (5.1)	41 (5.5)	9 (5.4)	22 (4.6)	2 (28.6)	3 (10.0)	21 (4.3)	

*HIV status*								
Positive	576 (29.8)	172 (22.9)	42 (25.2)	218 (45.1)	1 (14.3)	3 (10.0)	140 (28.3)	<0.001
Negative	1,096 (56.7)	499 (66.4)	102 (61.1)	191 (39.5)	6 (85.7)	22 (73.3)	276 (55.9)	
HIV test declined	261 (13.5)	81 (10.8)	23 (13.8)	74 (15.3)	0 (0.0)	5 (16.7)	nn (15.8)	

**Table 2:** Extrapulmonary site of infection distribution.

Extrapulmonary site	Frequency	Percentage
Abdominal Kochs	45	4.8
Pleural effusion	314	33.9
Spine	110	11.9
Disseminated	340	36.7
Lymph nodes	30	3.2
Meninges	40	4.3
Pericardial effusion	32	3.4
Others	16	1.7

**Table 3:** Demographic and clinical factors associated with TB mortality.

Parameters	Alive *N* (%)	Dead *N* (%)	Bivariate odds ratio (95% CI)	Multivariate odds ratio (95% CI)
Total population	956	483		

*Age at diagnosis*				
13–24	117 (12.2)	78 (16.1)	1.00	1.00
25–44	460 (48.1)	217 (44.9)	0.708 (0.509-0.983)	0.789 (0.560-1.112)
45–64	300 (31.4)	152 (31.5)	0.760 (0.537-1.075)	0.801 (0.557-1.152)
≥65	79 (8.3)	36 (7.5)	0.684 (0.420-1.113)	0.685 (0.412-1.159)

*Gender*				
Females	338 (35.4)	186 (38.5)	1.00	1.00
Males	618 (64.6)	297 (61.5)	0.873 (0.697-1.095)	0.914 (0.720-1.159)

*HIV status*				
Negative	629 (65.8)	191 (39.5)	1.00	1.00
Positive	218 (22.8)	218 (45.1)	3.293 (2.570-4.220)	3.261 (2.531-4.202)
Unknown/refused	109 (11.4)	74 (15.3)	2.336 (1.597-3.131)	2.168 (1.533-3.066)

*TB disease site*				
EPTB	418 (43.7)	225 (46.6)	1.00	1.00
Pulmonary TB	531 (55.5)	255 (52.8)	0.892 (0.716-1.112)	0.877 (0.623-1.234)
Not known	7 (0.7)	3 (0.6)	0.796 (0.204-3.109)	0.759 (0.183-3.156)

*Bacteriological status*				
Confirmed	352 (36.8)	146 (30.2)	1.00	1.00
Unconfirmed	554 (57.9)	303 (62.7)	1.319 (1.039-1.674)	0.981 (0.680-1.415)
Not documented	50 (5.2)	34 (7.0)	1.639 (1.018-2.640)	1.382 (0.842-2.271)

## Data Availability

The dataset analyzed during this study is available from the corresponding author on reasonable request.

## References

[1] WHO (2022). *Global tuberculosis report 2022*.

[2] World Health Organization (2016). Global tuberculosis report. https://www.afro.who.int/health-topics/tuberculosis-tb.

[3] Law I., Floyd K., the African TB Prevalence Survey Group (2020). National tuberculosis prevalence surveys in Africa, 2008–2016: an overview of results and lessons learned. *Tropical Medicine & International Health*.

[4] Chaisson R. E., Martinson N. A. (2008). Tuberculosis in Africa--combating an HIV-driven crisis. *The New England Journal of Medicine*.

[5] World Health Organization (2020). *Global tuberculosis report, 2019. WHO/CDS/TB/2019.15*.

[6] Bonsu F., Addo K. K., Alebachew Z. (2020). The international journal of tuberculosis and lung disease. *The Official Journal of the International Union against Tuberculosis and Lung Disease*.

[7] Ben Ayed H., Koubaa M., Marrakchi C. (2018). Extrapulmonary tuberculosis: update on the epidemiology, risk factors and prevention strategies. *International Journal of Tropical Disease*.

[8] World Health Organization *Definitions and reporting framework for tuberculosis – 2013 revision (updated 2014 and 2020)*.

[9] Mahajan N. K. (2020). Controversies and pitfalls in the diagnosis of extrapulmonary tuberculosis with a focus on genital tuberculosis. *US Endocrinology*.

[10] Parsons L. M., Somoskövi A., Gutierrez C. (2011). Laboratory diagnosis of tuberculosis in resource-poor countries: challenges and opportunities. *Clinical Microbiology Reviews*.

[11] Rimal R., Shrestha D., Pyakurel S. (2022). Diagnostic performance of GeneXpert MTB/RIF in detecting MTB in smear-negative presumptive TB patients. *BMC Infectious Diseases*.

[12] World Health Organization (WHO) (2011). *Rapid implementation of the Xpert MTB/RIF diagnostic test*.

[13] Li S., Liu B., Peng M. (2017). Diagnostic accuracy of Xpert MTB/RIF for tuberculosis detection in different regions with different endemic burden: a systematic review and meta-analysis. *PLoS One*.

[14] Kohli M., Schiller I., Dendukuri N. (2018). Xpert^®^ MTB/RIF assay for extrapulmonary tuberculosis and rifampicin resistance. *Cochrane Database Systematic Review*.

[15] WHO (2020). Global tuberculosis report: executive summary. https://apps.who.int/iris/handle/10665/337538.

[16] Jayasooriya S., Jobe A., Badjie S. (2019). The burden of non-TB lung disease presenting to TB clinics in the Gambia: preliminary data in the Xpert® MTB/Rif era. *Public Health Action*.

[17] Jayasooriya S., Dimambro-Denson F., Beecroft C. (2023). Patients with presumed tuberculosis in sub-Saharan Africa that are not diagnosed with tuberculosis: a systematic review and meta-analysis. *Thorax*.

[18] Chaaba E., Bwembya J., Nyambe E. (2023). Mortality among persons receiving tuberculosis treatment in Itezhi-Tezhi District of Zambia: a retrospective cohort study. *PLOS Global Public Health*.

[19] Adamu A. L., Gadanya M. A., Abubakar I. S. (2017). High mortality among tuberculosis patients on treatment in Nigeria: a retrospective cohort study. *BMC Infectious Diseases*.

[20] Uplekar M., Weil D., Lonnroth K. (2015). WHO's new end TB strategy. *The Lancet*.

[21] Ohene S. A., Bakker M. I., Ojo J., Toonstra A., Awudi D., Klatser P. (2019). Extra-pulmonary tuberculosis: a retrospective study of patients in Accra, Ghana. *PloS One*.

[22] Addo S. O., Mensah G. I., Mosi L. (2021). Trends in extrapulmonary TB cases at three teaching hospitals in Ghana, 2008-2017. *Public Health Action*.

[23] Chan-Yeung M., Noertjojo K., Chan S. L., Tam C. M. (2002). Sex differences in tuberculosis in Hong Kong. *The International Journal of Tuberculosis and Lung Disease*.

[24] WHO (2017). *Global Tuberculosis report*.

[25] Sandgren A., Hollo V., van der Werf M. J. (2013). Extrapulmonary tuberculosis in the European Union and European Economic Area, 2002 to 2011. *Eurosurveillance*.

[26] Hoogendoorn J. C., Ranoto L., Muditambi N. (2017). Reduction in extrapulmonary tuberculosis in context of antiretroviral therapy scale-up in rural South Africa. *Epidemiology and Infection*.

[27] Arega B., Mersha A., Minda A. (2020). Epidemiology and the diagnostic challenge of extra-pulmonary tuberculosis in a teaching hospital in Ethiopia. *PloS One*.

[28] Karstaedt A. S. (2013). Extrapulmonary tuberculosis among adults: experience at Chris Hani Baragwanath academic hospital, Johannesburg, South Africa. *South African Medical Journal*.

[29] Tedla E., Ayalew G., Mekonnen F. (2020). Mycobacterium tuberculosis burden, multidrug resistance pattern, and associated risk factors among presumptive extrapulmonary tuberculosis cases at Dessie Referral Hospital, Northeast Ethiopia. *The Egyptian Journal of Chest Diseases and Tuberculosis*.

[30] Alemu A., Yesuf A., Gebrehanna E. B. (2020). Incidence and predictors of extrapulmonary tuberculosis among people living with *human immunodeficiency virus* in Addis Ababa, Ethiopia: a retrospective cohort study. *PloS One*.

[31] Lisboa M., Fronteira I., Colove E., Nhamonga M., Martins M. . R. O. (2019). Time delay and associated mortality from negative smear to positive Xpert MTB/RIF test among TB/HIV patients: a retrospective study. *BMC Infectious Diseases*.

[32] Neshati H., Sheybani F., Naderi H. (2018). Diagnostic errors in tuberculous patients: a multicenter study from a developing country. *Journal of Environmental and Public Health*.

[33] García J. I., Mambuque E., Nguenha D. (2020). Mortality and risk of tuberculosis among people living with HIV in whom TB was initially ruled out. *Scientific Reports*.

[34] Mateyo K., Kerkhoff A. D., Dunn I., Nteeni M. S., Muyoyeta M. (2022). Clinical and radiographic characteristics of presumptive tuberculosis patients previously treated for tuberculosis in Zambia. *PLOS One*.

[35] Bello G., Faragher B., Sanudi L. (2017). The effect of engaging unpaid informal providers on case detection and treatment initiation rates for TB and HIV in rural Malawi (Triage Plus): a cluster randomised health system intervention trial. *PloS One*.

[36] Purohit M., Mustafa T. (2015). Laboratory diagnosis of extra-pulmonary tuberculosis (EPTB) in resource-constrained setting: state of the art, challenges and the need. *Journal of Clinical and Diagnostic Research: JCDR*.

[37] Bouton T. C., Forson A., Kudzawu S. (2019). High mortality during tuberculosis retreatment at a Ghanaian tertiary center: a retrospective cohort study. *The Pan African Medical Journal*.

[38] Moore D. A., Roper M. H. (2007). Diagnosis of smear-negative tuberculosis in people with HIV/AIDS. *The Lancet*.

[39] Ryu Y. J. (2015). Diagnosis of pulmonary tuberculosis: recent advances and diagnostic algorithms. *Tuberculosis and Respiratory Diseases*.

[40] Agbor A. A., Bigna J. J., Plottel C. S. (2015). Characteristics of patients co-infected with HIV at the time of inpatient tuberculosis treatment initiation in Yaoundé, Cameroon: a tertiary care hospital-based cross-sectional study. *Archives of Public Health*.

[41] Harries A. D., Hargreaves N. J., Gausi F., Kwanjana J. H., Salaniponi F. M. (2001). High early death rate in tuberculosis patients in Malawi. *The International Journal of Tuberculosis and Lung Disease*.

[42] Takarinda K. C., Sandy C., Masuka N. (2017). Factors associated with mortality among patients on TB treatment in the southern region of Zimbabwe, 2013. *Tuberculosis Research and Treatment*.

[43] Kebede W., Gudina E. K., Balay G., Abebe G. (2021). Diagnostic implications and inpatient mortality related to tuberculosis at Jimma Medical Center, southwest Ethiopia. *Journal of Clinical Tuberculosis and Other Mycobacterial Diseases*.

[44] Tola H. H., Tol A., Shojaeizadeh D., Garmaroudi G. (2015). Tuberculosis treatment non-adherence and lost to follow up among TB patients with or without HIV in developing countries: a systematic review. *Iranian Journal of Public Health*.

[45] Tadesse S. (2016). Stigma against tuberculosis patients in Addis Ababa, Ethiopia. *PloS One*.

[46] Alao M. A., Ibrahim O. R., Chan Y. H. (2022). Clinical and psychosocial determinants of patients with tuberculosis/human immunodeficiency virus co-infection: a structural equation model approach. *Nigerian Journal of Clinical Practice*.

[47] Santha T., Garg R., Frieden T. R. (2022). Risk factors associated with default, failure and death among tuberculosis patients treated in a DOTS programme in Tiruvallur District, South India, 2000. *The International Journal of Tuberculosis and Lung Disease*.

[48] Horne D. J., Royce S. E., Gooze L. (2010). Sputum monitoring during tuberculosis treatment for predicting outcome: systematic review and meta-analysis. *The Lancet Infectious Diseases*.

[49] Viana P. V. S., Paiva N. S., Villela D. A. M., Bastos L. S., de Souza Bierrenbach A. L., Basta P. C. (2020). Factors associated with death in patients with tuberculosis in Brazil: competing risks analysis. *PloS One*.

[50] Edessa D., Adem F., Hagos B., Sisay M. (2021). Incidence and predictors of mortality among persons receiving second-line tuberculosis treatment in sub-Saharan Africa: a meta-analysis of 43 cohort studies. *PloS One*.

